# Extracellular Adenosine Triphosphate Binding to P2Y1 Receptors Prevents Glutamate-Induced Excitotoxicity: Involvement of Erk1/2 Signaling Pathway to Suppress Autophagy

**DOI:** 10.3389/fnins.2022.901688

**Published:** 2022-06-07

**Authors:** Yiping Xiong, Duanyang Zhou, Kai Zheng, Wenchuan Bi, Yun Dong

**Affiliations:** ^1^School of Pharmaceutical Sciences, Health Science Center, Shenzhen University, Shenzhen, China; ^2^School of Pharmacy and Food Sciences, Zhuhai College of Science and Technology, Zhuhai, China

**Keywords:** neuroexcitotoxicity, autophagy, extracellular ATP, neuroprotection, Erk1/2 signaling pathway

## Abstract

Glutamate-induced neuroexcitotoxicity could be related to the pathophysiology of some neurodegenerative diseases including Parkinson’s disease and Alzheimer’s disease. Extracellular ATP exerts a wide variety of functions, such as attenuating Aβ-mediated toxicity, inhibiting *N*-Methyl-D-Aspartate (NMDA) receptor subunit combinations, and aggravating ischemic brain injury. However, the effect of extracellular ATP on glutamate-induced neuroexcitotoxicity remains largely unknown. Herein, we showed that extracellular ATP prevented the glutamate-induced excitotoxicity *via* binding to its P2Y1 receptors. We found that excessive glutamate triggered cellular reactive oxygen species (ROS) overproduction and mitochondrial membrane potential damage, which were significantly attenuated by extracellular ATP. Besides, glutamate activated autophagy, as illustrated by the increased protein level of autophagic marker LC3II and decreased level of p62, and glutamate-induced neuroexcitotoxicity could be completely abolished by autophagy inhibitor chloroquine. In addition, we revealed that extracellular ATP activated Erk1/2 signaling to suppress autophagy and to exert its neuroprotective effects, which was further reduced by autophagy agonist rapamycin and the selective Erk1/2 inhibitor PD0325901. Taken together, our findings suggest that extracellular ATP binding to P2Y1 receptors protected against glutamate-induced excitotoxicity *via* Erk1/2-mediated autophagy inhibition, implying the potential of ATP for the treatment of neurodegenerative disorders.

## Introduction

Neuroexcitotoxicity caused by glutamate is related to pathogenesis in a variety of neurodegenerative diseases, including Parkinson’s disease (PD) and Alzheimer’s disease (AD) (Yang et al., 2014; Sabogal-Guáqueta et al., 2019; Chen et al., 2020). Glutamate acts as an excitatory neurotransmitter and exert many functions in mammalian brain, such as learning and memory, *via* modulating Na^+^, Ca^2+^ and K^+^ influx through its several receptors (Ribeiro et al., 2017). Excessive glutamate release induces the overactivation of glutamate receptors and high levels of Ca^2+^ entry into neural cells, which in turn triggers excitotoxicity and ultimately neural death. Many *in vitro* experiments have demonstrated that glutamate at high concentrations induces neural cell death. For instance, glutamate resulted in an increase of mitophagy and high levels of zinc ion in HT-22 cells (Jin et al., 2018), or induced apoptosis in the rat primary cortical neurons (Hu et al., 2013) and SH-SY5Y cells (Xiong et al., 2021). Evidence showed that glutamate induced excitotoxicity by increasing reactive oxygen species (ROS) in cultured primary neurons (Kritis et al., 2015). Therefore, it is of importance to identify a mechanism that exerts neuroprotective effects against glutamate-induced neuroexcitotoxicity.

Adenosine triphosphate (ATP) has been reported to play important roles in the central nervous system (CNS). Massive extracellular release of ATP is involved in the etiopathology of some neurodegenerative disorders. For instance, extracellular ATP activated astrocytes to mediate motor neuron death (Gandelman et al., 2010). Extracellular ATP also contributed to photoreceptor neurodegeneration in rodents and induced intracellular alpha-synuclein to damage lysosome functions (Puthussery and Fletcher, 2009; Gan et al., 2015). However, increasing evidence has showed that extracellular ATP could be neuroprotective. An analogue of ATP, ATP-γ-S-(α, β-CH2), rescued PC12 cells from oxidative stress injury and amyloid beta-induced toxicity (Danino et al., 2015). Besides, extracellular ATP administration protected astrocytes against H_2_O_2_-induced oxidative injury (Shinozaki et al., 2005), or activated glutamate receptor to modulate synaptic plasticity in the hippocampus (Yamazaki and Fujii, 2015). Importantly, ATP has recently been demonstrated as an endogenous inhibitor of *N*-methyl-D-aspartate (NMDA) receptors against glutamate-induced mitochondrial membrane potential disruption in cultured rat hippocampal neurons (Fujikawa et al., 2012). Therefore, these actual results suggest that extracellular ATP acts opposite effects in the CNS: neuroprotection and neurodegeneration. Although ATP has been reported to be neuroprotective against glutamate, the contribution of extracellular ATP on excitotoxicity induced by glutamate and its underlying mechanism in SH-SY5Y neuroblastoma cells still maintain largely unknown.

In the present study, we investigated the potential of extracellular ATP on glutamate-induced excitotoxicity and explored the underlying molecular mechanisms in SH-SY5Y cells. We clearly demonstrated that extracellular ATP suppress autophagy to attenuate glutamate-induced excitotoxicity *via* Erk1/2 signaling. Our study therefore highlighted that ATP could be as an effective agent in the treatment of some neurodegenerative diseases.

## Materials and Methods

### Reagents and Antibodies

Extracellular ATP, PD0325901, rapamycin, and chloroquine (CQ) were purchased from MedChemExpress (MCE, San Rafael, CA, United States), and L-glutamate was obtained from Sigma–Aldrich (St. Louis, MO, United States). MRS2500 tetraammonium salt was purchased from Tocris (Bio-techne, Shanghai, China). Dulbecco’s modified Eagle’s medium (DMEM), fetal bovine serum (FBS), penicillin/streptomycin, trypsin and phosphate-buffered saline (PBS) were purchased from Gibco (Thornton, NSW, Australia). The Cell Counting kit (CCK-8) was purchased from Dojindo Laboratory (Kumamoto, Japan). The JC-1 assay kit (5,50,6,60-tetrachloro-1,10,3,30-tetraethyl-imidacarbocyanine iodide) and RIPA lysis buffer were obtained from Beyotime Biotech (China). The ROS assay kit (DCFH-DA) was purchased from Meilun (China). The FITC-labeled Annexin V Apoptosis Detection kit was purchased from BD Biosciences (Canada). The antibodies of p-Erk1/2, Erk1/2, p-Akt, Akt, LC3, SQSTM1/p62, GAPDH, FITC and horseradish peroxidase (HRP)-conjugated goat anti-rabbit/mouse antibody were obtained from Cell Signaling Technology (Danvers, MA, United States). The second antibody conjugated anti-mouse IgG and conjugated anti-rabbit IgG were purchased from Thermo. Anti-P2Y1 receptor antibody was purchased from Alomone Labs (Jerusalem BioPark, Israel).

### Cell Culture and Treatment

Human neuroblastoma cell SH-SY5Y cells were purchased from The Global Bioresource Center (ATCC, United States) and were cultured in DMEM with the addition of 10% FBS, 100 U/mL penicillin and 100 U/mL streptomycin at 37°C in a humidified atmosphere of 95% air and 5% CO2 incubator. SH-SY5Y cells were plated at a density of 2 × 10^4^/well in 96-well plates and 2 × 10^5^/well in 6-well plates for 24 h. Thereafter, SH-SY5Y cells were pretreated with either PBS or extracellular ATP followed by a stimulation of 10 mM glutamate for 24 h and experimental analyses, such as cell viability, mitochondrial membrane potential, ROS generation and protein expression, were performed.

### Cell Viability

Cell viability was quantified by CCK-8 kit following the manufacturer’s instruction. After SH-SY5Y cells seeded in 96-well plates were treated with extracellular ATP and/or glutamate for 24 h, 10 μL CCK-8 solution was added into each well and incubated at 37°C for 1 h. Thereafter, the absorbance at 450 nm was determined using a microplate reader (BioTek).

### Measurement of Intracellular Reactive Oxygen Species

The intracellular levels of reactive oxygen species (ROS) were measured with probe 2′,7′-dichlorodihydrofluorescein diacetate (DCFH-DA, ROS assay kit). Briefly, the treated SH-SY5Y cells were washed twice with PBS and incubated in serum-free medium with 10 μM DCFH-DA at 37°C for 30 min. Fluorescent images were captured in six different areas of each well under a fluorescent microscope (AxioVert A1, Zeiss, Germany) and measured using Image-J v1.8.0 software. The percentage of ROS-positive cells was calculated.

### Measurement of Mitochondrial Membrane Potential

The level of mitochondrial membrane potential was measured by a commercial cyanine JC-1 assay kit, either as red fluorescent J-aggregates or as green fluorescent J-monomers, as previous (Dong et al., 2020). J-aggregates at higher mitochondrial concentrations reflect higher membrane potential whereas J-monomers at lower mitochondrial concentrations indicate lost membrane potential. Accordingly, fluctuation of mitochondrial membrane potential is represented as the J-aggregate/J-monomer (red/green) fluorescence intensity ratio. The treated SH-SY5Y cells were washed with PBS and then incubated with 1 mL JC-1 solution (5 μM) at 37°C for 30 min. Subsequently, images of the cells were captured in six different areas of each well under the fluorescence microscope. The changes in mitochondrial membrane potential were presented as the red/green fluorescence intensity ratio using Image-J v1.8.0 software.

### Immunofluorescence

After each treatment, SH-SY5Y cells were fixed with 4% paraformaldehyde for 15 min. After twice washing, the cells were permeabilized with 0.5% Triton X-100 and blocked with 5% BSA. Cells were incubated with LC3 antibody or SQSTM1/p62 antibody (1:1,000) in blocking solution for 24 h and then incubated with FITC conjugated anti-mouse IgG or with FITC conjugated anti-rabbit IgG (1:2,000) for 2 h. Thereafter, DAPI (1:1,000, Beyotime, China) was incubated with cells in PBS solution for 10 min at room temperature in dark. Samples were observed under an inverted fluorescence microscope (AxioVert A1, Zeiss, Germany). The cells in six different areas of each well were photographed under fluorescent microscope. Images were analyzed with Image-J v1.8.0 software. The percentage of each group was calculated as LC3-positive or p62-positive cell number/total cell number × 100%.

### Western Blot

After each treatment, cells were rinsed twice with cold PBS and lysed by RIPA lysis buffer containing proteases inhibitors (1:50, Roche, Germany), phosphatase inhibitors 1 and 2 (1:100, Roche, Germany), phenylmethanesulfonyl fluoride (PMSF, 1:100, Beyotime, China), and nuclease (1:1,000, Biolong Biotech, China) on ice. The collected cell lysates were then vortexed and the insoluble cell debris were removed by centrifugation at 12,000 × *g* for 10 min at 4°C. The total protein concentrations were measured using a Pierce BCA Protein Assay Kit (Thermo Fisher Scientific, Waltham, MA, United States). The equal amount of protein extracts (10 μg) was separated on a 10–15% sodium dodecyl sulfate-polyacrylamide (SDS-PAGE) gel and transferred onto PVDF membranes (Bio-Rad, Hercules, CA, United States). Membranes were blocked with 5% milk in Tris-buffered saline containing 0.05% (v/v) Tween 20 (TBS-T) at room temperature for 60 min and then incubated with the relevant primary antibodies at 4°C overnight. The antibodies were diluted as follows: p-Erk1/2, Erk1/2, LC3II, p62, Akt and p-Akt and GAPDH in 1:1,000 dilution. Afterward, membranes were incubated with corresponding HRP-conjugated secondary antibodies at a 1:5,000 dilution for 60 min at room temperature. Reactive bands were visualized by the Quantity One automatic imaging analysis system (Bio-Rad, Hercules, CA, United States) using enhanced chemiluminescence ECL (Millipore, Kankakee, IL, United States). Finally, the protein band intensities were calculated using Image-J v1.8.0 software.

### Statistical Analysis

Statistical comparisons were performed using one-way analysis of variance (ANOVA) following by *a post hoc multiple-comparison Tukey* test. All quantified biological data were expressed as means ± standard error of the mean (SEM) of at least three independent experiments. Mean values were considered to be significant at *P < 0.05, ^**^P < 0.01, and ^***^P < 0.001.

## Results

### Extracellular Adenosine Triphosphate Reduces Glutamate-Mediated Excitotoxicity *via* Binding to P2Y1 Receptors in SH-5Y5Y Cells

**FIGURE 1:**
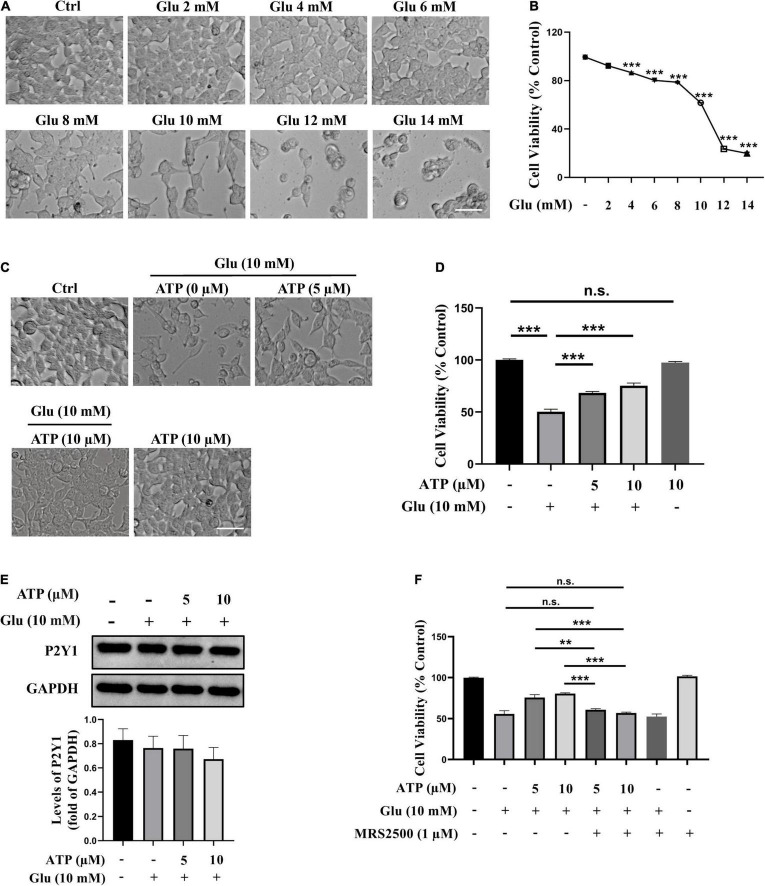
Extracellular adenosine triphosphate (ATP) prevents glutamate-induced excitotoxicity *via* binding P2Y1 receptors in SH-SY5Y cells. **(A)** Representative images of the cell morphology after cultured SH-SY5Y cells were treated with various concentrations of glutamate (Glu) for 24 h; the control group (Ctrl) consisted of the untreated cells. Scale bar = 100 μm under 20× magnification. **(B)** Cell viability was detected using Cell Counting kit (CCK-8). **(C)** Representative images of the cell morphology after pretreated by ATP (5 and 10 μM) or phosphate-buffered saline (PBS) following by the application of 10 mM glutamate for 24 h. Scale bar = 100 μm under 20× magnification. **(D)** Cell viability was calculated. **(E)** The protein expressions of P2Y1 receptor, and the bar chart presented the levels of P2Y1 receptor in all groups. Expression of GAPDH was served as a loading control. **(F)** CCK-8 kit was performed to evaluated cell viability after the administration of MRS2500, and the changes were shown in histograms as percentage with control. All data represent mean ± SEM from at least three independent experiments. **P < 0.01 and ***P < 0.001 versus the glutamate group or the ATP treated control group. n.s., no significance.

To study the vulnerability of SH-SY5Y cells to glutamate, SH-SY5Y cells were initially exposed to several concentrations of glutamate (2, 4, 6, 8, 10, 12, and 14 mM) for 24 h, and the cell viability was detected using CCK-8 kit. The result showed that glutamate caused a dose-dependent decrease in cell viability as compared to control (Figures 1A,B). Glutamate at 4 mM led to a significant decrease in cell density (*P* < 0.001). Glutamate of 10 mM could induce almost half SH-SY5Y cell loss and thus was applied in all the following experiments. We then examined whether extracellular ATP exerted neuroprotective effect against glutamate-induced excitotoxicity in SH-SY5Y cells as that in rat hippocampal neurons. SH-SY5Y cells were incubated with 10 mM glutamate in the presence or absence of 5 μM or 10 μM ATP for 24 h, respectively. As shown in Figures 1C,D, extracellular ATP at both concentrations significantly prevented glutamate-induced reduction of cell viability (*P* < 0.001, compared to the glutamate group), while 10 μM extracellular ATP did not exhibit any cytotoxicity. In addition, extracellular ATP remarkably prevented reduction of EdU incorporation induced by glutamate (Supplementary Figure 1). Importantly, we demonstrated that purinergic P2Y1 receptors of extracellular ATP were expressed in SH-SY5Y cells, and the expression of P2Y1 was not affected by all treatments (Figure 1E). However, MRS2500, the selective antagonist of P2Y1 receptors, completely abolished the neuroprotection of extracellular ATP (Figure 1F). Finally, we tested whether glutamate triggered apoptosis in SH-SY5Y cells and no apoptotic cells were observed (Supplementary Figure 2), in accordance with the previous study (Brasil et al., 2021). Taken together, these results demonstrated that extracellular ATP protects SH-SY5Y cells from glutamate-mediated excitotoxicity *via* the P2Y1 receptors.

### Extracellular Adenosine Triphosphate Reduces Intracellular Reactive Oxygen Species Overproduction and Mitochondrial Membrane Potential Damage

**FIGURE 2:**
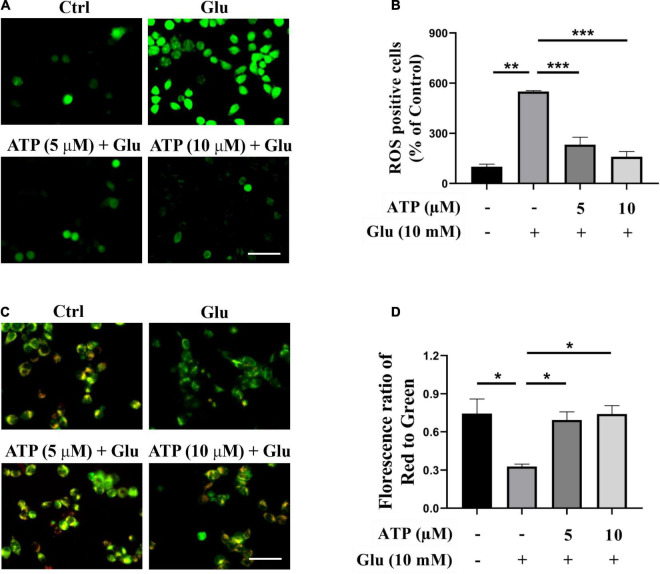
Extracellular ATP reduces reactive oxygen species (ROS) overgeneration and restored mitochondrial membrane potential functions induced by glutamate. Cultured SH-SY5Y cells were incubated in different concentrations (5 and 10 μM) of extracellular ATP, following by the application of 10-mM glutamate for 24 h; the control group consisted of the untreated cells. **(A)** SH-SY5Y cells were stained with DCFH-DA. Covered glasses with cells were observed under a fluorescent microscope. Scale bar = 100 μm under 20 × magnification. **(B)** Fluorescent density mean of ROS level was calibrated. **(C)** JC-1 staining in SH-SY5Y cells. Scale bar = 100 μm under 20× magnification. **(D)** The ratio of the red/green fluorescence optical density were analyzed by image J. All data in bar charts present mean ± SEM from at least three independent experiments. *P < 0.05, **P < 0.01, and ***P < 0.001 versus the glutamate group or the control group.

Previous studies have manifested that glutamate induced excessive ROS productions and mitochondrial dysfunction to cause cell death (Atlante et al., 2001; Xu et al., 2012). Accordingly, we analyzed intracellular ROS production induced by glutamate in the presence or absence of extracellular ATP. As shown in Figures 2A,B, ROS production was significantly induced by 10 mM glutamate, which was further prevented by either 5 or 10 μM extracellular ATP. We also monitored the mitochondrial membrane potential by JC-1 staining, and found that the red/green ratio was significantly reduced by 10 mM glutamate compared to the control group (Figures 2C,D). However, administration of extracellular ATP substantially increased the red/green ratio (*P* < 0.001, compared to the glutamate group), suggesting that extracellular ATP could prevent mitochondrial membrane potential disruption. Therefore, extracellular ATP exerted neuroprotective effects by reducing ROS production and restoring mitochondrial functions.

### Extracellular Adenosine Triphosphate Neuroprotects Against Glutamate-Induced Excitotoxicity *via* Suppressing Autophagy

**FIGURE 3:**
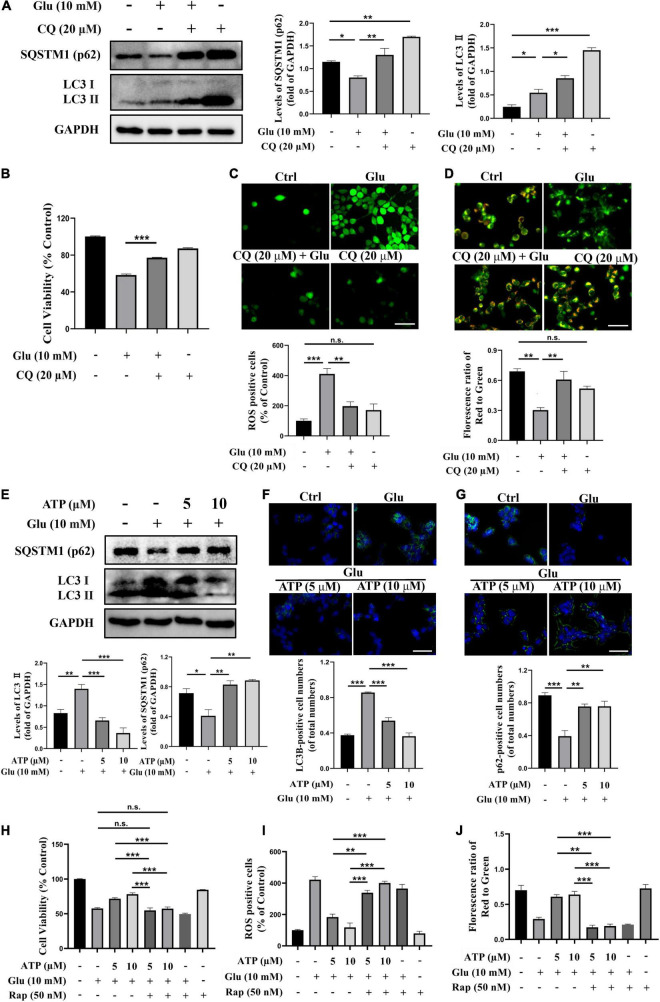
Extracellular ATP prevents glutamate-induced excitotoxicity *via* suppressing autophagy in SH-SY5Y cells. **(A)** The protein expression of LC3 and p62 were determined by western blot in SH-SY5Y cells treated with 10-mM Glu in the presence or absence of chloroquine (CQ) (20 μM), and expression of GAPDH was served as loading control. The quantitation of LC3 and p62 expressions were calibrated. **(B)** Cell viability was evaluated. **(C)** Treated SH-SY5Y cells were stained with DCFH-DA, and the changes were shown in histograms as percentage with control. Scale bar, 100 μm under 20× magnification. **(D)** Treated SH-SY5Y cells were stained using JC-1, and the changes were shown in histograms as percentage with control. Scale bar, 100 μm under 20× magnification. **(E)** The protein expressions of LC3 and p62 were determined by western blot in SH-SY5Y cells treated with 10-mM Glu in the presence of ATP (5 and 10 μM), and expression of GAPDH was served as loading control. The quantitation of LC3II and p62 expressions was calibrated. **(F)** LC3II and **(G)** QSTM1/p62 detected using immunocytochemistry were photographed under fluorescent microscope, and images were analyzed. **(H)** Cell viability was detected. **(I)** ROS-positive cells were calibrated. **(J)** Treated SH-SY5Y cells were stained using JC-1, and the changes were presented. All data in bar charts present mean ± SEM from at least three independent experiments. *P < 0.05, **P < 0.01, ***P < 0.001 versus the ATP treated group or the glutamate group. n.s., no significance.

There were numerous evidence showing the connections among mitochondrial damage, ROS overproduction and autophagy (Scherz-Shouval and Elazar, 2011; Li et al., 2015). To address whether autophagy was involved in glutamate-induced excitotoxicity in SH-SY5Y cells, the protein level of LC3II, a critical marker of autophagy, and the level of SQSTM1/p62 (p62), a marker for autolysosomal degradation, were analyzed. Apparently, exposure to glutamate significantly increased the level of LC3II compared to the control group (*P* < 0.001) whereas the expression of p62 was downregulated (Figure 3A), suggesting that glutamate activated autophagy process. Treatment with chloroquine (CQ), an autophagic inhibitor, further upregulated the amount of LC3II and p62, indicating the activation of autophagy by glutamate (Figure 3A). Furthermore, CQ treatments significantly increased cell viability and prevented ROS overproduction induced by glutamate (Figures 3B,C). Administration of CQ also significantly ameliorated the mitochondrial membrane potential disruptions (Figure 3D). Therefore, these above results clearly demonstrated that glutamate activates autophagy to cause excitotoxicity. Importantly, we found that extracellular ATP restored the activated autophagy by glutamate, as evidenced by decreased expression of LC3II and upregulated expression of p62 (Figure 3E). The immunofluorescence results showed glutamate-induced increased LC3II expression and decreased p62 expression were significantly prevented by extracellular ATP (Figures 3F,G). These suggested that ATP not only deceased autophagosome accumulation and suppressed autolysosomal degradation. Consistently, when cells were simultaneously pretreated with extracellular ATP as well as rapamycin, an agonist of autophagy, the protective effects of ATP against glutamate-induced excitotoxicity disappeared (Figures 3H–J and Supplementary Figure 3). CQ did not significantly increase ATP neuroprotection against glutamate-induced excitotoxicity (Supplementary Figure 4). Taken together, these results suggested that extracellular ATP suppresses autophagy to inhibit glutamate-induced excitotoxicity.

### Erk1/2 Signaling Pathway Is Involved in the Neuroprotective Effects of Extracellular Adenosine Triphosphate

**FIGURE 4:**
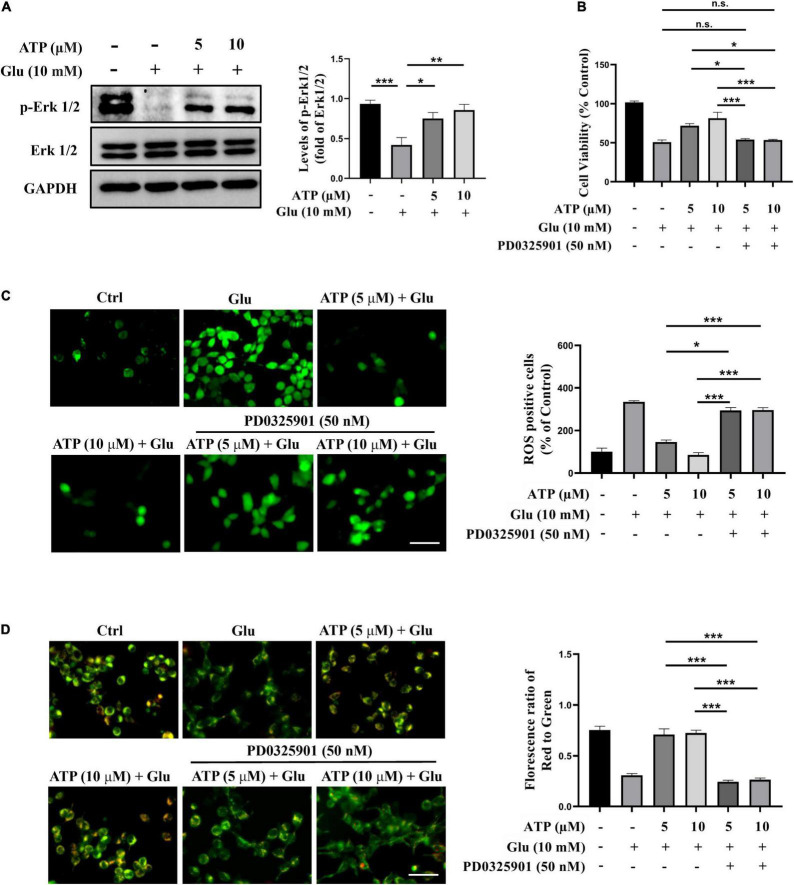
The neuroprotective effects of extracellular ATP against glutamate involves Erk1/2 signaling pathway. Cultured SH-SY5Y cells were treated with 10 mM Glu in the presence or absence of ATP (5 and 10 μM). The control group consists of the untreated cells. **(A)** The protein expression of p-Erk1/2 and Erk1/2 were determined by western blotting, and the quantitation of p-Erk1/2 and Erk1/2 expressions were calibrated. Expression of GAPDH was served as loading control. **(B)** CCK-8 kit was performed to evaluated cell viability. **(C)** All treated SH-SY5Y cells were stained with DCFH-DA, and fluorescent density mean of ROS level was calibrated. Scale bar = 100 μm under 20× magnification. **(D)** All treated SH-SY5Y cells were stained with JC-1 kit, and the ratio of the red/green fluorescence optical density were analyzed. Scale bar = 100 μm under 20× magnification. All data in bar charts present mean ± SEM from at least three independent experiments. *P < 0.05, **P < 0.01, ***P < 0.001 versus the ATP treated group or the glutamate group. n.s., no significance.

Next, we examined the possible roles of several signaling pathways implicated in the ATP-mediated protective response to glutamate-induced neuroexcitotoxicity, such as PI3K/Akt and Erk1/2 signaling pathways (Ali et al., 2018; Chen et al., 2020; Liu et al., 2021). We monitored the protein levels of phosphorylated Erk1/2 (p-Erk1/2) and phosphorylated Akt (p-Akt), and found that the levels of p-Erk1/2 were down-regulated by glutamate in SH-SY5Y cells; whereas the treatments of extracellular ATP significantly increased the expression of p-Erk1/2 (Figure 4A), suggesting that ATP activates Erk1/2 to antagonize glutamate. However, the expression of p-Akt was not affected by extracellular ATP and glutamate (Supplementary Figures 5E,F). To further explore the potential of Erk1/2 signaling in ATP neuroprotection against glutamate-mediate excitotoxicity, PD0325901, a selective Erk1/2 inhibitor, was employed. As shown in Figure 4B, the enhancement of cell viability treated by extracellular ATP against glutamate was totally abolished by PD0325901. In addition, PD0325901 prevented the decrease of ROS generation treated by ATP (Figure 4C), as well as inhibited the ATP-mediated recovery of mitochondrial membrane potential (Figure 4D). Furthermore, PD0325901 prevented the ATP-promoted increase in EdU incorporation (Supplementary Figures 5C,D). Together, these results suggested that the activated Erk1/2 pathway is involved in the neuroprotective effects of extracellular ATP against glutamate-induced excitotoxicity.

### Extracellular Adenosine Triphosphate Suppresses Autophagy *via* Erk1/2 Signaling Pathway

**FIGURE 5:**
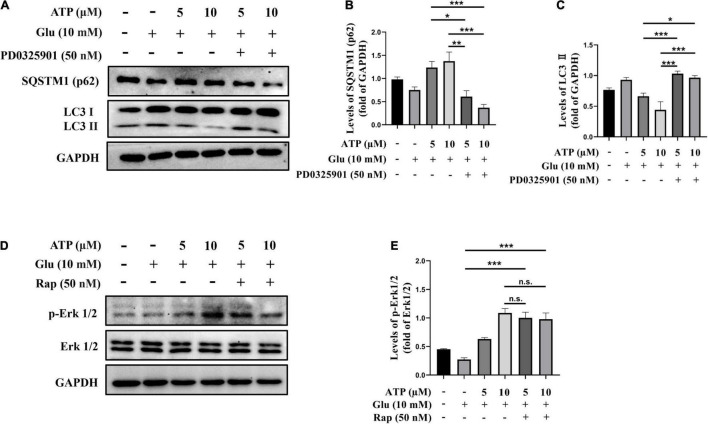
Upregulated p-Erk1/2 by extracellular ATP prevents autophagy induced by glutamate. Cultured SH-SY5Y cells were treated with or without ATP (5 and 10 μM), followed by the application of glutamate (10 mM) in the presence or absence of PD0325901 (50 nM) or rapamycin (50 nM) for 24 h; the untreated cells was the control group. **(A)** The expressions of LC3 and p62 were determined by western blot. Expression of GAPDH served as loading control. The quantitation of **(B)** p62 expression and **(C)** LC3 expression were calibrated. **(D)** The expressions of p-Erk1/2 and Erk1/2 were determined by western blot. Expression of GAPDH served as loading control. **(E)** The quantitation of p-Erk1/2 expression was calibrated. All data in bar charts present mean ± SEM from at least three independent experiments. *P < 0.05, **P < 0.01 and ***P < 0.001 versus the glutamate group or the ATP treated group. n.s., no significance.

To further definite the neuroprotective mechanism of extracellular ATP against glutamate, we examined the connection between Erk1/2 and autophagy inhibition. SH-SY5Y cells were treated with glutamate and ATP in the presence or absence of PD0325901, and protein levels of LC3II levels and p62 were analyzed. Apparently, PD0325901 treatment entirely reversed the expression levels of LC3II and p62 mediated by extracellular ATP (Figures 5A–C). However, autophagy inducer rapamycin did not influence the expression of p-Erk1/2 affected by extracellular ATP (Figures 5D,E). Therefore, we concluded that extracellular ATP activates Erk1/2 signaling pathway to inhibit glutamate-mediated activation of autophagy.

## Discussion

Increasing studies have reported that extracellular ATP could be neuroprotective in the CNS. For instance, extracellular ATP could attenuate NMDA-mediated neurotoxicity in cultured hippocampal neurons by inhibiting the heterologous expression of NMDA receptors (Ortinau et al., 2003). Extracellular ATP regulated amyloid precursor protein processing to inhibit Aβ production (Chan et al., 2008). Enhancement of ATP production could ameliorate cognitive dysfunctions in a PD mouse model (Haga et al., 2019). In current study, we demonstrated that extracellular ATP protected SH-SY5Y cells from glutamate-induced excitotoxicity *via* activating Erk1/2 to suppress lethally autophagy. These studies suggest that extracellular ATP might be able to develop as a therapeutic strategy for neurodegenerative disorders.

Neuroexcitotoxicity caused by high concentration of glutamate is one of pivotal pathological features in neurodegenerative disorders (Hugon et al., 1996; Binvignat and Olloquequi, 2020). Abnormally high concentration of glutamate stimulates the excessive activation of *N*-methyl-D-aspartate (NMDA) subtype of ionotropic L-glutamate receptors, subsequently results in an excessive Ca^2+^ influx, which is taken up by mitochondria and subsequently triggers excitatory biochemical processes and death pathways (Rothstein, 1996; Jia et al., 2015). Furthermore, glutamate-mediated excitotoxicity involves ROS overproduction that leads to neuroinflammation and synaptic dysfunctions in rat brain regions (Khan et al., 2021). In the present study, we also demonstrated that glutamate remarkably caused ROS generation in SH-SY5Y cells (Figures 2A,B), which was consistent with previous studies that glutamate caused oxidative damage in SH-SY5Y cells (Sun et al., 2010; Lee et al., 2019). Massive ROS production aggressed mitochondria and led to mitochondrial dysfunctions (Sinha et al., 2013; Angelova and Abramov, 2018). Our previous study has shown that excessive ROS caused the loss of mitochondrial membrane potential and activated mitochondria-dependent cell deficits in HT-22 cells (Dong et al., 2020). Besides, ROS overproduction stimulated by glutamate could result in lipid peroxidation and mitochondrial dysfunctions to the detriment of cellular growth (Lee et al., 2019). Kushnareva et al. (2005) reported that excessive glutamate caused a reduction of mitochondrial ATP and an imbalance of homeostasis that precede commitment to neuronal death. Consistent with previous studies, we observed that exposure to 10 mM glutamate for 24 h indeed induced disruptions of mitochondrial membrane potential in SH-SY5Y cells (Figures 2C,D). Furthermore, we showed that 10 mM glutamate prevented cell proliferation by EdU staining (Supplementary Figure 1). Finally, glutamate induced HT-22 cell ferroptosis mediated by massive ROS production (Jiang et al., 2020). However, our data showed that glutamate could neither affect glutathione peroxidase 4 levels nor glutathione expression, which suggested that glutamate-induced excitotoxicity in SH-SY5Y cells was not related to ferroptosis (data not shown). Flow cytometer analysis showed that glutamate could not induce SH-SY5Y cell apoptosis (Supplementary Figure 2). Characteristics of cell injury in response to glutamate shown in these studies suggest that glutamate-induced excitotoxicity is associated to multiple molecular mechanisms. Together, we confirmed that glutamate induced excessive ROS generation, triggered mitochondrial dysfunctions, and prevented cell proliferation.

Adenosine triphosphate, a well-known intracellular energy currency, is a signaling molecule that influences cell proliferation, differentiation, survival, myelination as well as repairment in the CNS (Fields and Burnstock, 2006; Burnstock, 2007). Extracellular ATP not only counteracted H_2_O_2_-promoted cell death in astrocytes (Shinozaki et al., 2006), but also ameliorated cognitive impairments in a mouse model with PD (Haga et al., 2019). Notably, extracellular ATP has been implicated to act two opposite roles when it combines and activates different purine receptors (P2X receptors and P2Y receptors) in ischemic brain injury: pathogenesis and neuroprotection. For instance, P2 × 1, P2 × 2, P2 × 4, P2 × 7 receptors participated in the pathogenesis of cerebral ischemia whereas P2Y1 receptor could induce neuroinflammatory responses to neuroprotect against ischemic neuronal injury (Koizumi et al., 2007; Tu and Wang, 2009; Fukumoto et al., 2019). These studies demonstrated that the activation of different purine receptors could be crucial for extracellular ATP neuroprotection. Our current study elucidated that extracellular ATP protected SH-SY5Y cells from glutamate-induced neuroexcitotoxicity. The statistical results showed that several concentrations of extracellular ATP (2, 5, 10, 20, and 40 μM) could significantly rescue SH-SY5Y cells from 10 mM glutamate-mediated excitotoxicity, including increasing cell viability (Supplementary Figure 1A). The neuroprotective effects of extracellular ATP at 10, 20, and 40 μM were almost similar, and 2-μM ATP could not significantly prevent glutamate-induced cell loss. Therefore, extracellular ATP at 5 and 10 μM were applied the following experiments. The observation further showed that ATP not only inhibited ROS generation but also recovered mitochondrial functions (Figure 2). Importantly, we found that P2Y1 receptors expressed in SH-SY5Y cells, and its selective antagonist, MRS2500, absolutely eliminated neuroprotective effects of extracellular ATP against glutamate (Figures 1E,F), which was supported by a previous study that activated P2Y1 receptors were neuroprotective (Fukumoto et al., 2019). Evidence, on the contrary, demonstrated that P2Y1 receptor blockade could normalized activation of astrocytes and then attenuated cognitive dysfunctions in AD mouse models (Reichenbach et al., 2018), indicating that activation of P2Y1 receptor could mediate neurodegeneration. The opposite roles of P2Y1 receptors in neurodegeneration and neuroprotection might be due to the activation of P2Y1 receptors in different neural cells: neuroprotection in neuronal cells and neurodegeneration in glial cells. Evidence showed that P2Y2 receptor is believed to be neuroprotective under inflammatory conditions (Weisman et al., 2012). To explore whether ATP prevented glutamate-induced neuroexcitotoxicity by its binding to P2Y2 receptors, diquafosol tetrasodium, the agonist of P2Y2 receptor, was applied. Our data showed that diquafosol tetrasodium could not affect glutamate-mediated excitotoxicity in SH-SY5Y cell, indicating that the neuroprotective effects of extracellular ATP were not related to P2Y2 receptor (data not shown). In neurons, both ATP and its secondary metabolite ADP turn to AMP through CD39 or E-NTPDase and then is hydrolyzed into adenosine by CD73 or 5′ nucleotidase. Adenosine is widely valued in the treatment of the CNS diseases (Liu et al., 2019). In order to verify ATP neuroprotective effects, ADP was applied to pretreat SH-SY5Y cells incubated by glutamate, and CCK-8 assay showed that ADP did not rescue SH-SY5Y cells from glutamate-induced excitotoxicity (data not shown). Taken together, we concluded that extracellular ATP binding to its P2Y1 receptors protected from glutamate-induced excitotoxicity in SH-SY5Y cells.

Under normal physiological conditions, autophagy maintains neuronal homeostasis and neuronal survival (Mizushima et al., 2008). Autophagy has been implicated to play a dominant role in neurodegenerative disorders, such as AD and PD. Autophagy could promote the metabolism of Aβ and the assembling of tau, and thus dysfunctions of autophagy might lead to the progress of AD (Zhu et al., 2013; Li et al., 2017). Moreover, autophagy processes could alter PD-associated genes, like SNCA, GBA, ATP13A2, and FBXO7 (Lu et al., 2020). These studies suggested that selective agonists of autophagy might be developed as potential drugs for neurodegenerative disorders. However, excessive autophagy induces oxidative damage, neuroinflammatory injury, and then exacerbates neurodegeneration. Evidence has revealed that high concentrations of glutamate induced autophagy *via* the activation of the lysosomal Ca^2+^ channels TPC1 and TPC2 in neural cells (Pereira et al., 2017). In this study, we found that 10 mM glutamate significantly induced an increase of LC3II and a decrease of p62/SQATM1 (Figure 3A), indicating that autophagy could be involved in glutamate-induced excitotoxicity. An inhibitor of autophagy, CQ, significantly suppressed glutamate-induced excitotoxicity, including increasing cell viability, decreasing ROS generation, and recovering mitochondrial functions (Figures 3B–D). Our results demonstrated that glutamate-induced excitotoxicity was mediated by autophagy, which was supported by a previous study (Pereira et al., 2017). Importantly, the treatments of ATP were able to suppress autophagy induced by glutamate (Figure 3 and Supplementary Figure 3). A study demonstrated that knockdown or pharmacological inhibition of purinergic P2Y12 receptors suppressed autophagy process in advanced atherosclerosis (Pi et al., 2021), which supported that extracellular ATP could suppress autophagy processes. Similarly, high concentrations of adenosine, one of ATP metabolites, inhibited autophagy in β cells (Israeli et al., 2018). Opposite to our study, a literature showed that extracellular ATP could be though P2 × 7 receptors to trigger autophagy in primary microglia (Fabbrizio et al., 2017). In summary, these studies implied that extracellular ATP could exert different functions through activating different receptors in a certain condition. In the current study, we confirmed that the neuroprotective effects of ATP against glutamate-induced excitotoxicity was *via* preventing autophagy.

Multiple mechanisms were involved in the neuroprotective effects of extracellular ATP. For instance, extracellular ATP binding to P2Y1 receptors protected pancreatic duct epithelial cells *via* cAMP signaling pathway (Seo et al., 2016). Extracellular ATP binding to P2Y13 purinergic receptors could neuroprotect astrocytes *via* PI3K/Akt/GSK3 pathway (Carrasquero et al., 2009). Also, extracellular ATP attenuated stroke *via* inhibiting glutamate release to neuroprotection of antioxidants against (Hurtado et al., 2003). Our current study demonstrated extracellular ATP neuroprotected against glutamate-mediated excitotoxicity through Erk1/2 signaling pathway in SH-SY5Y cells (Figure 4). PD0325901, a selective antagonist of Erk1/2 remarkably abolished the neuroprotective effects of extracellular ATP. Consistently, activation of Erk1/2 promoted PC12 cell survival by blocking the expression of the pro-apoptotic factor, BH3-only protein Bim (Terasawa et al., 2009). A previous study showed that activated ATP-dependent potassium channels promoted glioma cell proliferation by upregulated Erk1/2 signaling pathway (Huang et al., 2009), which supported our findings that extracellular ATP pretreatment *via* Erk1/2 signaling pathway promoted SH-SY5Y cell proliferation (Supplementary Figure 5). Interestingly, extracellular ATP induced the S-phase cell cycle arrest of oral squamous cell carcinoma SAS cells *via* its binding to P2Y1 receptors-mediated Erk1/2 signaling (Lau et al., 2021). This research showed the antitumor effects of extracellular ATP were through interaction with P2Y1 receptors to promote Erk1/2 signaling, which was opposite to its effects in SH-SY5Y cells shown in the current study. This might be due to extracellular ATP exerted multiple functions in different microenvironments. Notably, our results showed that extracellular ATP though upregulation of p-Erk1/2 expression prevented autophagy against glutamate excitotoxicity (Figure 5). Similarly, Sun et al. (2019) demonstrated that active MEK1 protected against H_2_O_2_-induced autophagy though Erk1/2 signaling pathway in neonatal rat cardiac ventricular cardiomyocytes. Evidence, on the contrary, demonstrated that activation of Erk1/2 could contribute to glutamate-induced oxidative injury in primary cortical neurons (Stanciu et al., 2000; Sun et al., 2019). Another study also showed that PD98059, a blockade of Erk1/2 pathway, suppressed H_2_O_2_-induced cell death (Bhat and Zhang, 1999). These studies illustrate that Erk1/2 signaling pathway can promote either neuronal survival or neuronal death under different paradigms. One possible explanation is that the magnitude and duration of Erk1/2 activation determines the different downstream effectors exerting the death-promoting capacity and cellular outcomes (Sun et al., 2019).

The current research is only based on non-clinical *in vitro* experiments, and a combination of *in vivo* and *in vitro* experiments is still needed to further explore the role of extracellular ATP as a potential candidate against excitotoxicity.

## Conclusion

We clearly demonstrated that extracellular ATP binding to P2Y1 receptors prevented glutamate-mediated neuroexcitotoxicity through activating Erk1/2 to suppress lethally autophagy in SH-SY5Y cells (Supplementary Figure 6). Our present findings provide new insights into the neuroprotective molecular mechanism of extracellular ATP and extend the therapeutic strategy of neurodegenerative diseases.

## Data Availability Statement

The original contributions presented in the study are included in the article/Supplementary Material, further inquiries can be directed to the corresponding author.

## Author Contributions

YX performed most of the experiments. YD conceived the project and designed the experiments. DZ, KZ, and WB participated in data analysis. All authors contributed to the article and approved the submitted version.

## Conflict of Interest

The authors declare that the research was conducted in the absence of any commercial or financial relationships that could be construed as a potential conflict of interest.

## Publisher’s Note

All claims expressed in this article are solely those of the authors and do not necessarily represent those of their affiliated organizations, or those of the publisher, the editors and the reviewers. Any product that may be evaluated in this article, or claim that may be made by its manufacturer, is not guaranteed or endorsed by the publisher.
