# Supplementary material for: Extracellular Adenosine Triphosphate Binding to P2Y1 Receptors Prevents Glutamate-Induced Excitotoxicity: Involvement of Erk1/2 Signaling Pathway to Suppress Autophagy
Source: Front Neurosci. 2022 Jun 7;16:901688. doi: 10.3389/fnins.2022.901688 (PMC9209742; doi:10.3389/fnins.2022.901688)
Supplement: Supplementary file 1 [file Data_Sheet_1.docx]

Extracellular ATP binding to P2Y1 receptors prevents glutamate-induced excitotoxicity: involvement of Erk1/2 signaling pathway to suppress autophagy

SUPPLEMENTARY MATERIAL

**Analysis of Apoptosis**

Cells were seeded onto 12-well plates for 24 h and then treated with various concentration of glutamate for another 24 h. After treatment, collected the cells of each group separately and wash them twice with cold PBS. Thereafter, the cells were resuspended in 500 µl 1× binding buffer, containing Annexin V/FITC and 5 µl PI for 15 min at room temperature in the dark. Detection of apoptosis was evaluated using Annexin V-FITC/PI apoptosis detection kit (BD Biosciences, Mississauga, ON, Canada) following the manufacturers’ instruction, which was conducted immediately after preparation of samples in the dark. Cell apoptosis was analyzed using a flow cytometer. Data were analyzed using FACSAria equipped with the CellQuest Software.

**Measurement of Cell Proliferation**

EdU incorporation into SH-SY5Y cells were measured by using EdU cell proliferation Kit with Alexa Fluor 488 (Beyotime, China) following the manufacturer’s instructions. Briefly, cells were incubated with 10 µM EdU solution in DMEM for 4 h. Thereafter, cells were washed twice with washing buffer (PBS containing 3% BSA), followed by fixation of 4% polyformaldehyde for 15 min and then permeabilization with PBS containing 0.3% Triton X-100 for 20 min. After another twice washing, cells were incubated with azide-conjugated Alexa Fluor 495 for 30 min in click addictive reactive buffer with 4 mM CuSO4. DAPI (1:1,000, Beyotime, China) was incubated with cells in PBS solution for 10 min at room temperature in dark. Cells were then washed three times with washing buffer prior to observation. The cells in six different areas of each well were photographed under an inverted fluorescent microscope. Images were analyzed with Image-J v1.8.0 software. The percentage of proliferated cells was calculated as EdU-positive cell number/total cell number × 100%.

**
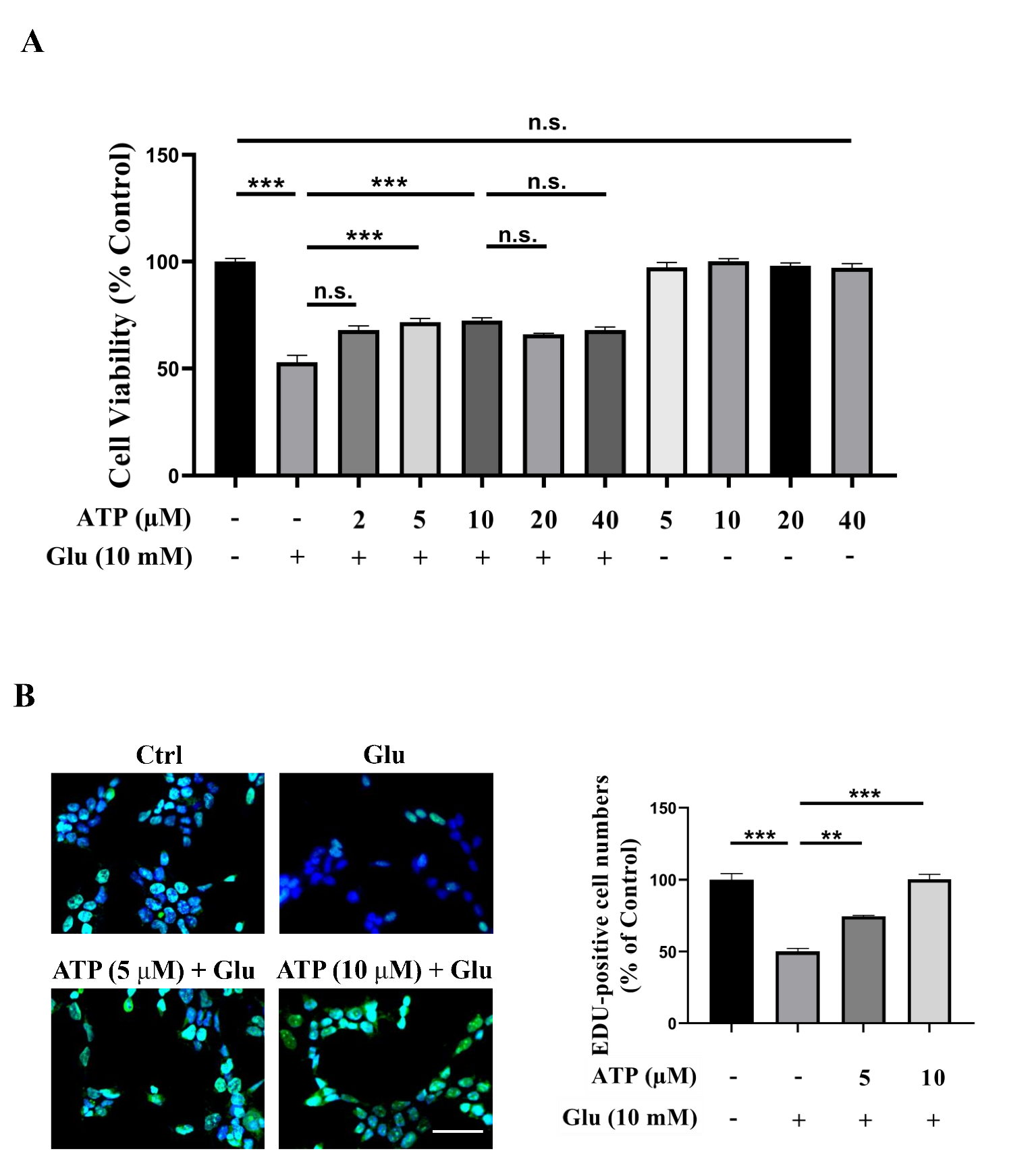
**

**Supplementary Figure 1. Extracellular ATP protects SH-SY5Y cells against glutamate-induced excitotoxicity.** (**A**) Different concentrations (2, 5, 10, 20, 40 μM) of extracellular ATP were pretreated following by application of 10 mM glutamate for 24 h, and cell viability was calculated with CCK-8 kit. (**B**) Representative images and quantitative measurement of EdU incorporation to cells after all treatments. Scale bar, 100 µm. All data in the bar chart represent mean ± SEM from at least three independent experiments. ** *P* < 0.01, *** *P* < 0.001 versus the glutamate group.


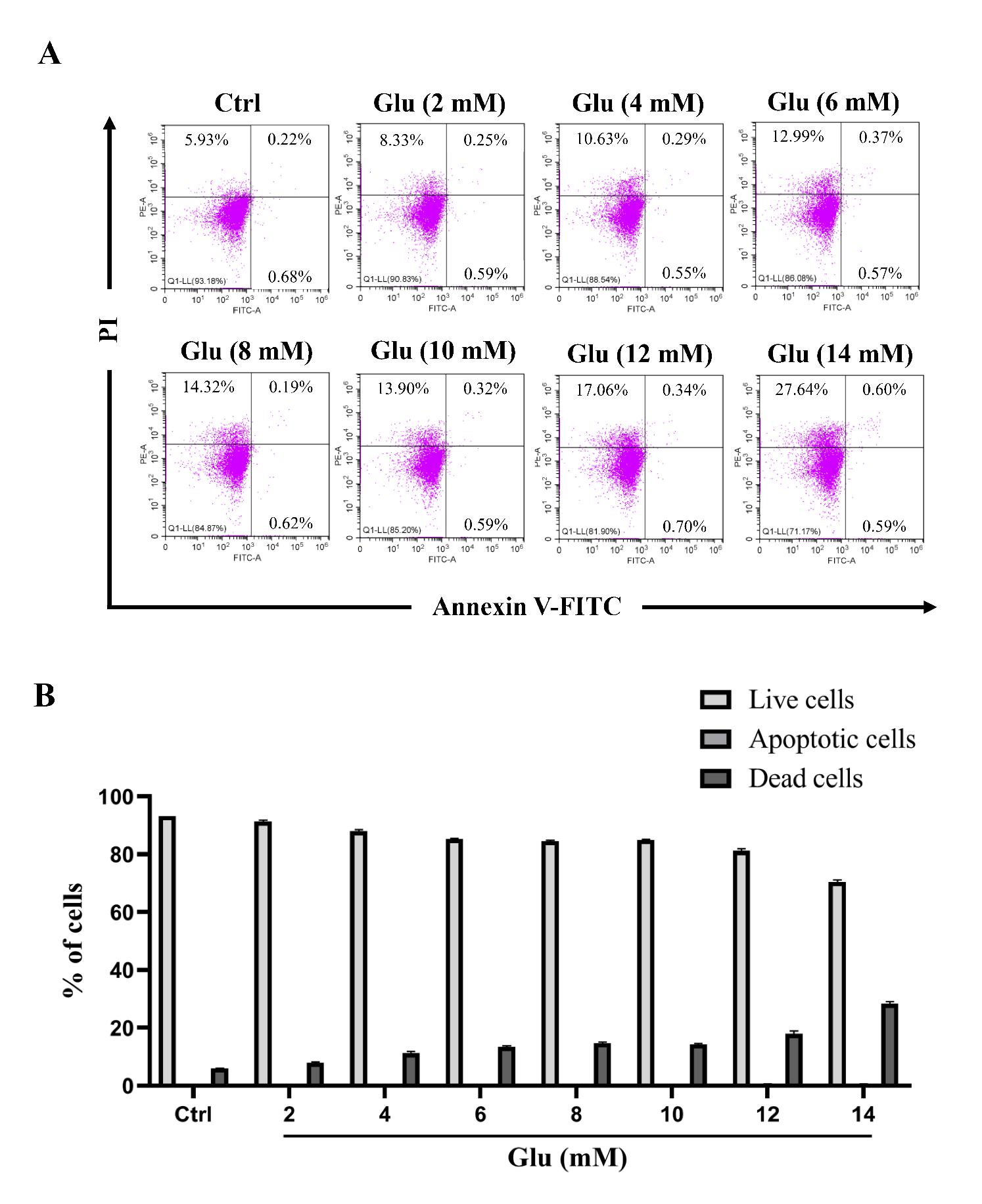


**Supplementary Figure 2. Glutamate at different concentrations does not trigger apoptosis in SH-SY5Y cells.** Cultured SH-SY5Y cells were treated with different concentrations of glutamate (2, 4, 6, 8, 10, 12, and 14 mM) for 24 h. (**A**) cell apoptosis was detected by flow cytometry with FITC-Annexin V/PI apoptosis detection Kit using a flow cytometer. (**B**) The percentage of apoptotic cell rates of live cells, apoptotic cells, and dead cells, as calibrated. All data in bar charts present mean ± SEM from at least three independent experiments.


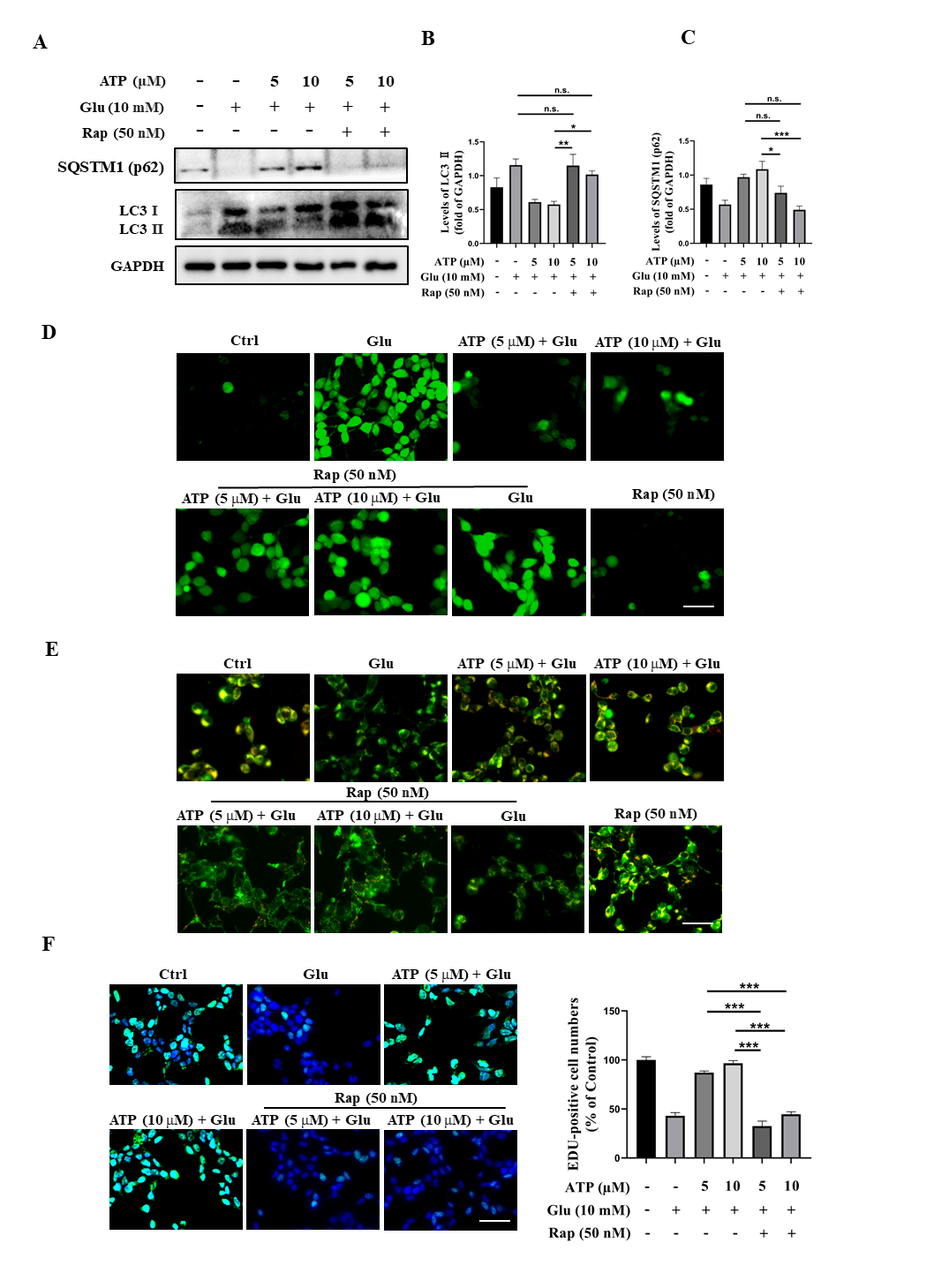


**Supplementary Figure 3.** **Rapamycin prevented the neuroprotective effects of extracellular ATP against glutamate.** Cultured SH-SY5Y cells were treated with or without glutamate (10 mM) and followed by the application of ATP (5 and 10 µM) in the presence or absence of rapamycin (50 nM) for 24 h. (**A**) The protein expression of LC3 and p62 were determined by western blot in SH-SY5Y cell, and expression of GAPDH served as loading control. (**B** and **C)** The quantitation of LC3 and p62 expressions were calibrated. (**D**) Treated SH-SY5Y cells were stained with DCFH-DA. (**E**) Treated SH-SY5Y cells were stained using JC-1. (**F**) Measurement of EdU incorporation to cells after all treatments, and the changes were shown in histograms as percentage. Scale bar, 100 μm. All data in bar charts present mean ± SEM from at least three independent experiments. * *P* < 0.05, ** *P* < 0.01, *** *P* < 0.001 versus the ATP treated group. n.s., no significance. Rap, rapamycin.


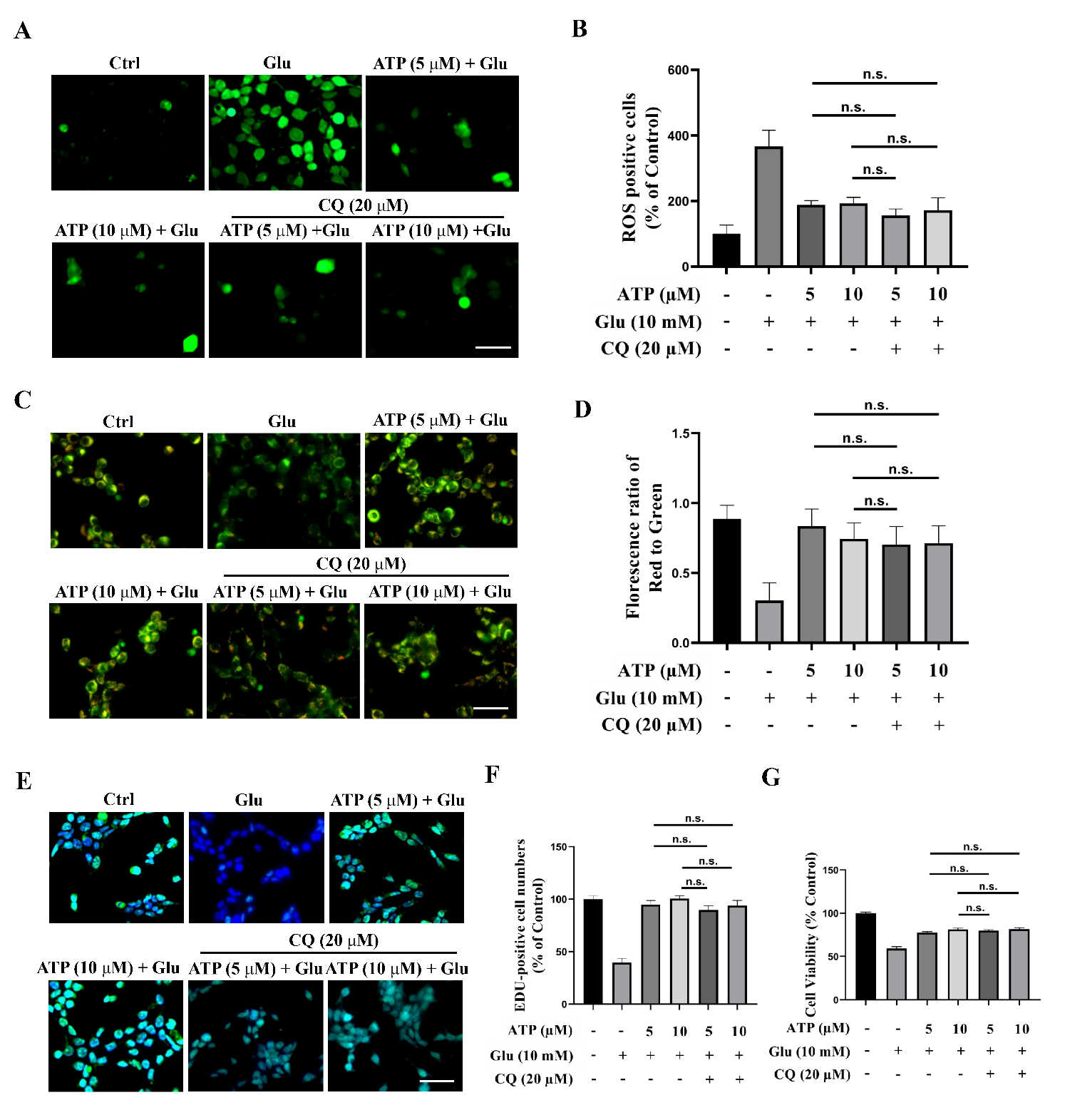


**Supplementary Figure 4.** **CQ does not influence the neuroprotective effects of extracellular ATP against glutamate-induced excitotoxicity.** Cultured SH-SY5Y cells were treated with or without Glu (10 mM) and followed by the application of ATP (5 and 10 µM) in the presence or absence of CQ (an antagonist of autophagy, 20 μM) for 24 h. (**A**) Treated SH-SY5Y cells were stained with DCFH-DA, and (**B**) the changes were shown in histograms as percentage. (**C**) Treated SH-SY5Y cells were stained using JC-1, and (**D**) the changes were shown in histograms as percentage. (**E** and **F**) Measurement of EdU incorporation to cells after all treatments. (**G**) Cell viability was calculated with CCK-8 kit. All data in bar charts present mean ± SEM from at least three independent experiments. ** *P* < 0.01 and *** *P* < 0.001 versus the ATP treated group or control group. n.s., no significance.


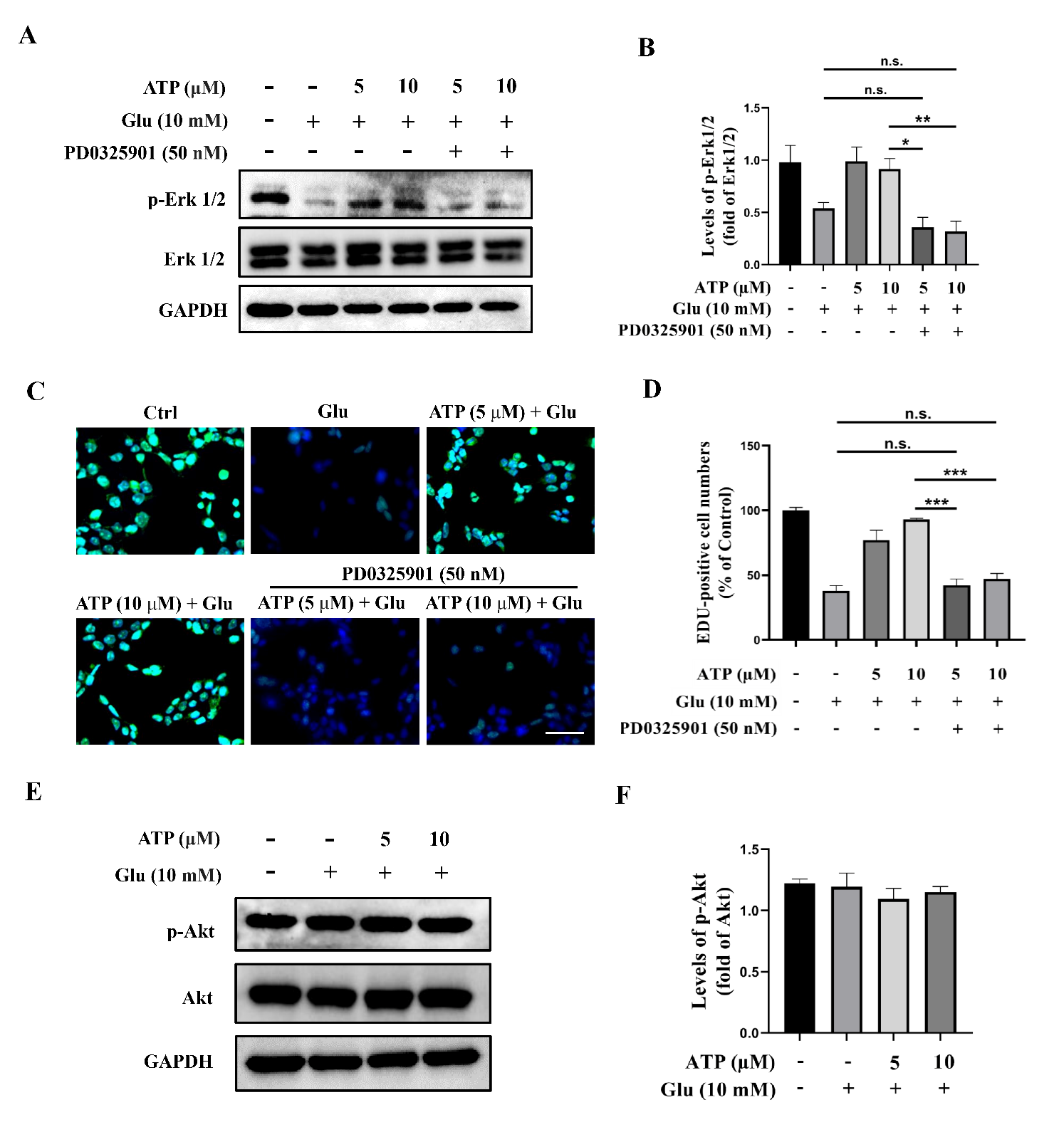


**Supplementary Figure 5. Erk1/2 signaling pathway but not PI3K/Akt was involved in extracellular ATP neuroprotection against glutamate-induced excitotoxicity.** (**A**) The expressions of p-Erk1/2 and Erk1/2 were determined by western blot after cultured SH-SY5Y cells were treated with glutamate (Glu) in the presence or absence of PD0325901 (an inhibitor of Erk1/2, 50 nM) for 24 h. Expression of GAPDH served as loading control. (**B**) The quantitation of p-Erk1/2 and Erk1/2 expressions were calibrated. (**C** and **D)**) Measurement of EdU incorporation to cells after all treatments. Scale bar = 100 μm under 20 × magnification. (**E**) The protein expression of p-Akt and Akt were determined by western blot in SH-SY5Y cell, and expression of GAPDH served as loading control. (**F**) The quantitation of p-Akt and Akt expressions were calibrated. All data in bar charts present mean ± SEM from at least three independent experiments. * *P* < 0.05, ** *P* < 0.01, ** *P* < 0.01. n.s., no significance.


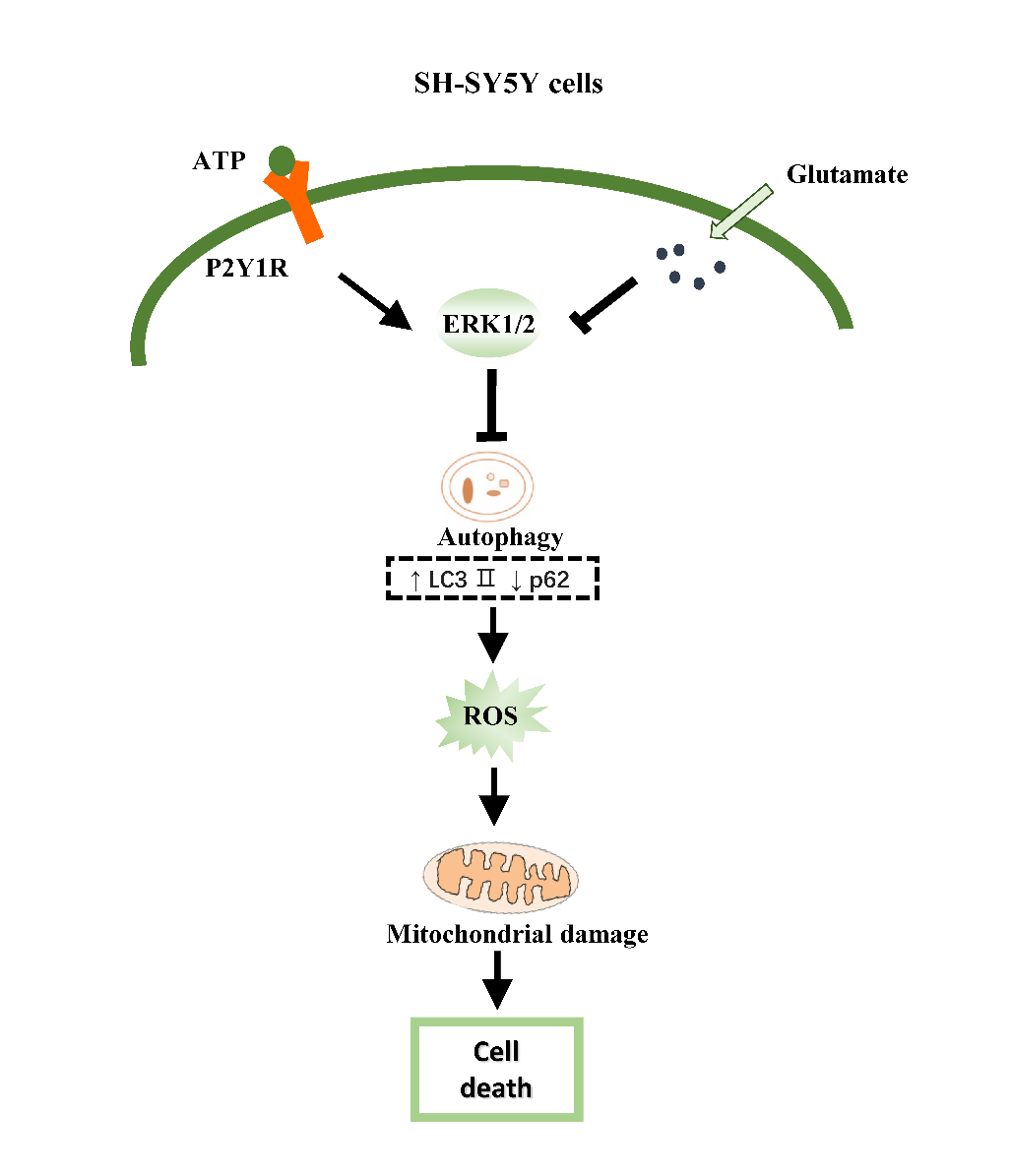


**Supplementary Figure 6. The schematic diagram of the neuroprotection of extracellular ATP against glutamate-induced excitotoxicity in SH-SY5Y cells.**
